# Homemade fenestration and chimney techniques for the left subclavian artery revascularization during zone 2 thoracic endovascular aortic repair

**DOI:** 10.3389/fcvm.2023.1144751

**Published:** 2023-05-31

**Authors:** Jiacheng Ye, Yuliang Li, Yue Lu, Yongzheng Wang, Bin Liu, Haiyang Chang

**Affiliations:** ^1^ Department of Intervention Medicine, The First Hospital of Nanping Affiliated to Fujian Medical University, Nanping, China; ^2^ Department of Intervention Medicine, The Second Hospital of Shandong University, Jinan, China; ^3^ Interventional Oncology Institute of Shandong University, Jinan, China

**Keywords:** aortic dissection, endovascular therapy, fenestration, chimney, left subclavian artery, zone 2 thoracic endovascular aortic repair

## Abstract

**Background:**

To investigate the safety and efficacy of homemade fenestration and chimney techniques for the left subclavian artery (LSA) revascularization during zone 2 thoracic endovascular aortic repair (TEVAR).

**Methods:**

From February 2017 to February 2021, 41 patients undergoing fenestration technique (group A) and 42 patients undergoing chimney technique (group B) to preserve the LSA during zone 2 TEVAR were enrolled in the present study. The procedure was indicated for dissections with unsuitable proximal landing zone with refractory pain and hypertension, rupture and malperfusion, and high-risk radiographic features. The baseline characteristics, peri-procedure, and follow-up clinical and radiographic data were recorded and analyzed. The primary endpoint was clinical success, and the secondary endpoints were rupture-free survival, LSA patency, and complications. Aortic remodeling, defined as patency, partial and complete thrombosis of the false lumen, was also analyzed.

**Results:**

Technical success was achieved in 38 and 41 patients in groups A and B, respectively. Four intervention-related deaths were confirmed, two in each group. Immediate post-procedural endoleaks were detected in two and three patients in group A and B, respectively. No other major complications were found in either group, except for one retrograde type A dissection in group A. During follow-up, the initial clinical success rates were 90.24% and 92.86% in groups A and B, respectively. The primary and secondary mid-term clinical success rates were 87.5% and 90% in group A, and both of them were 92.68% in group B. Rupture-free survival and LSA patency were not significantly different between the two groups. The incidence of complete thrombosis in the aorta distal to the stent graft was 67.65% and 61.11% in groups A and B, respectively.

**Conclusions:**

Apart from the lower clinical success rate of fenestration technique, both physician-modified techniques are available for LSA revascularization during zone 2 TEVAR and significantly promote favorable aortic remodeling.

## Introduction

Thoracic endovascular aortic repair (TEVAR) is routinely accepted as the first-line therapeutic option for type B aortic dissections (TBADs) with a lower incidence of morbidity and mortality than open surgery (1, 2). Despite the application of TEVAR has extended from the descending thoracic aorta to arch pathologies, an increasing risk of posterior circulation and upper extremity ischemia is considered to be associated with coverage of the left subclavian artery (LSA) during zone 2 TEVAR (3, 4). A meta-analysis reported that stroke has been a common finding after TEVAR, especially with LSA coverage without revascularization (5). TEVAR for thoracic aortic pathologies without a healthy proximal landing zone remains a challenge. Therefore, several commercially available devices and physician-modified techniques, including single-branched stent-graft, fenestration, and chimney techniques, have been introduced for LSA revascularization during zone 2 TEVAR (6–10).

According to previous studies, the issue of fenestrated endograft integrity may be related to long-term outcomes (8, 11), and the chimney technique is considered to increase the risk of endoleaks (12, 13). Therefore, selection criteria for different physician-modified techniques for LSA revascularization during zone 2 TEVAR for TBADs have not been established. In the present study, we aimed to summarize our experience and evaluate the safety and efficacy of the fenestration and chimney techniques for LSA revascularization during zone 2 TEVAR.

## Materials and methods

### Patient enrollment

**Figure 1:**
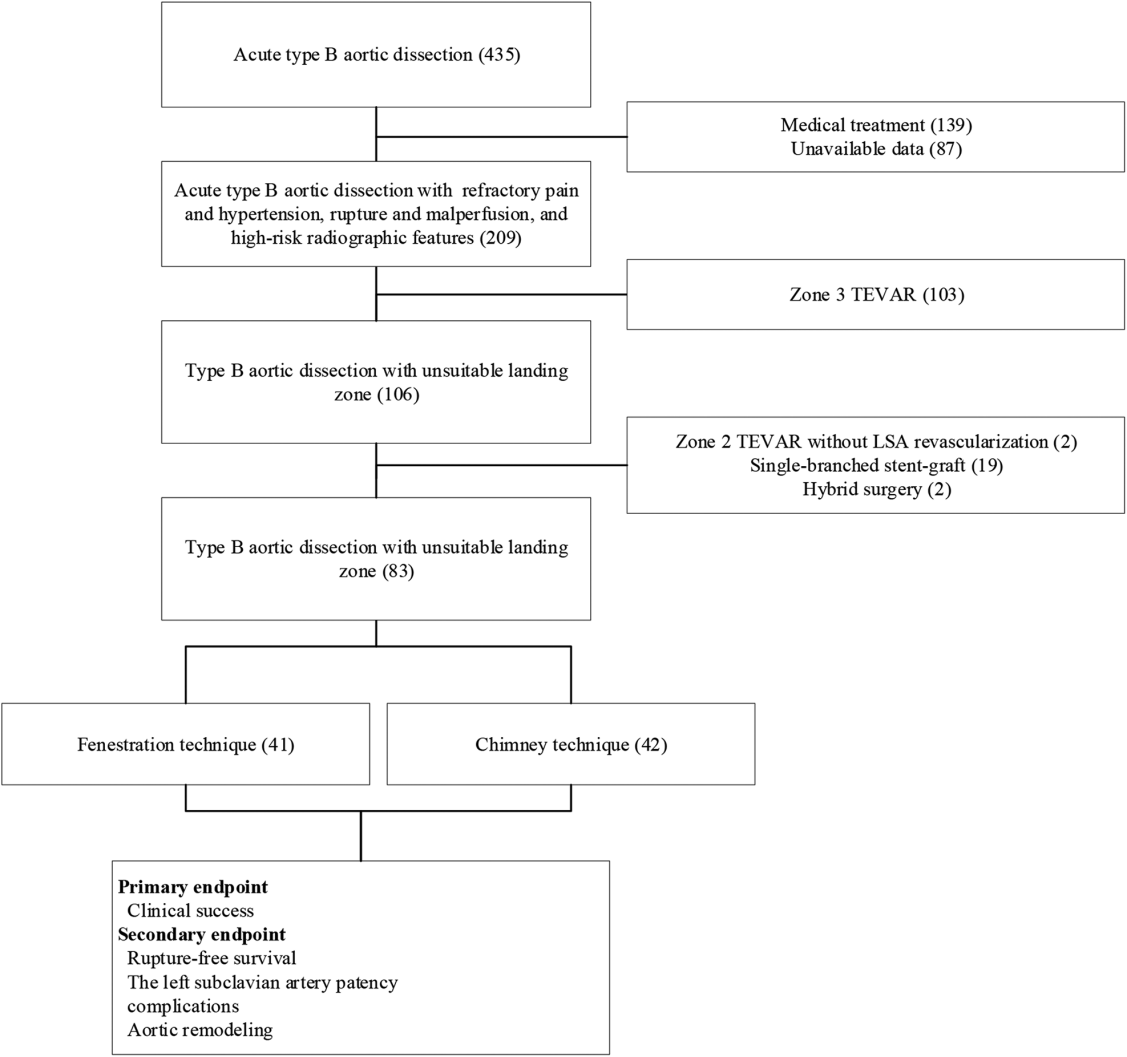
Flowchart of patient enrollment.

From February 2017 to February 2021, 41 patients who underwent the fenestration technique (group A) and 42 patients who underwent the chimney technique (group B) for LSA revascularization during zone 2 TEVAR for TBADs with unsuitable proximal landing zones (entry tear located distal <15 mm to the ostium of the LSA and dissection or intramural hematoma extending proximal to the LSA) were enrolled in this study. The present study was approved by our institutional review board, and the requirement for written informed consent was waived owing to the retrospective design of the study. The indications for TEVAR included recurrent/refractory pain (*n* = 55), visceral/renal/limb ischemia (*n* = 6), hypotension/aortic rupture (*n* = 15), and rapid aortic expansion (*n* = 7). Both techniques were offered without preference, and the patients decided which to undergo. Data related to demographic characteristics and in-hospital and follow-up clinical and radiographic outcomes were recorded and analyzed. A flowchart of patient enrollment is shown in Figure 1.

### Outcome criteria and definitions

The primary and secondary outcome criteria included the prevention of rupture or significant enlargement of the false lumen (aortic growth >5 mm per year), and death related to the primary pathology and the interventions. Technical success was defined as successful access to the arterial system using a remote site and deployment of the stent-graft at the intended location, absence of a type I or III endoleak and patent endoluminal graft without severe stenosis. TEVAR performed with the absence of type I or III endoleaks, significant enlargement of the false lumen or rupture, conversion to open repair, and death due to the original pathology and management was considered as clinical success. Leak at the proximal or distal graft attachment site, and around a fenestration or chimney stent was defined as type I, and Leak associated with modular disconnect or apposition failure, and fabric tear was considered to be type III. The stent-graft patency was defined as the stenosis should be <50% and the mean pressure gradient should be <10 mmHg (1, 2, 14). Major complications were defined as the requirement for significant re-intervention, prolongation of convalescence, and association with permanent disability and death (15).

### Radiographic data evaluation and procedure performance

With the assistance of Endosize software (Therenva SAS, Rennes, France), the perioperative and follow-up radiographic images were evaluated by the same two interventional radiologists with >15 years of experience in TEVAR, and who performed the procedure for all patients.

The fenestration and chimney techniques were performed under general anesthesia with tracheal intubation in all patients. Additionally, cerebrospinal fluid drainage was performed in two patients with the requirement to extend the distal landing zone in group B. Fenestration and chimney techniques were performed according to previous reports (7, 16). An Ankura stent-graft (Life-tech Scientific Co., Ltd., Shenzhen, China) was deployed to exclude the entry tear, and a Zilver bare metal stent (Cook Medical, Bloomington, IL, USA) was selected as chimney stent to preserve the LSA.

### Fenestration technique

**Figure 2:**
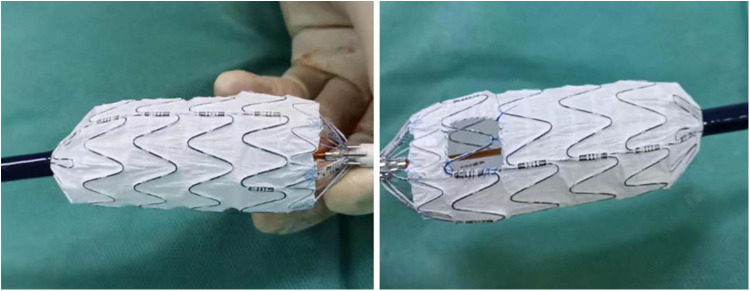
The fenestration was created in line with the radio-opaque middle-8-marker of the stent graft.

A 6 Fr sheath (Terumo Corporation, Tokyo, Japan) was inserted in the left brachial artery (LBA), and angiography was performed via a calibrated pigtail catheter (Cook Medical) advanced into the ascending aorta through the 6 Fr sheath. Subsequently, the proximal end of the stent graft (Life-tech Scientific) was unsheathed on the table, and the fenestration was created in linear alignment with the “8”-shaped radio-opaque marker, and a smooth edge was achieved by suturing circularly (Figure 2). Clock position was used to determine the LSA position on the reconstructed image. A 4 Fr tapered catheter (Cordis Corporation, Miami, USA) along with a 150 cm guidewire was advanced into the ascending aorta via surgically exposed common femoral artery (CFA). An extra stiff guidewire (Cook Medical) was exchanged for better support. Heparin (80 U/kg) was administrated intravenously. Subsequently, the modified stent graft was delivered to the aortic arch along with the extra-stiff guidewire (Cook Medical), and deployed with systolic blood pressure <90 mmHg, and transient apnea. Minor orientation of the stent graft was performed to indicate the accurate position of the fenestration once the first segment was released. Furthermore, a 4 Fr tapered-angle catheter was advanced into the LSA via the 6 Fr sheath to validate the patency of the LSA. An 8/10 mm × 40 mm bare metal stent (Cook Medical) was used to keep the LSA perfusion for those with unintentional covered LSA.

### Chimney technique

A 6 Fr sheath (Terumo Corporation) was deployed in the LBA percutaneously. Subsequently, a 5 Fr pigtail catheter (Cook Medical) was advanced over a guidewire into the ascending aorta for angiography. A unilateral CFA was exposed surgically. An extra-stiff guidewire (Cook Medical) was advanced into the ascending aorta along with a 4 Fr tapered catheter. The stent graft (Life-tech Scientific) was advanced into the aortic arch along with the extra stiff guidewire. Heparin (80 U/kg) was administrated intravenously, and the stent graft (Life-tech Scientific) was deployed proximal to the LSA and distal to the left common carotid artery under transient apnea with a systolic blood pressure ≤100 mmHg. A stiff guidewire (Abbott Medical) was exchanged over the pigtail catheter (Cook Medical), and an 8/10 mm × 40/60 mm bare self-expanded stent (Cook Medical) was introduced parallel to the main stent graft to keep the LSA patent. The proximal segment protruded to the aortic lumen ≥20 mm with the distal end remaining in the LSA.

**Figure 3:**
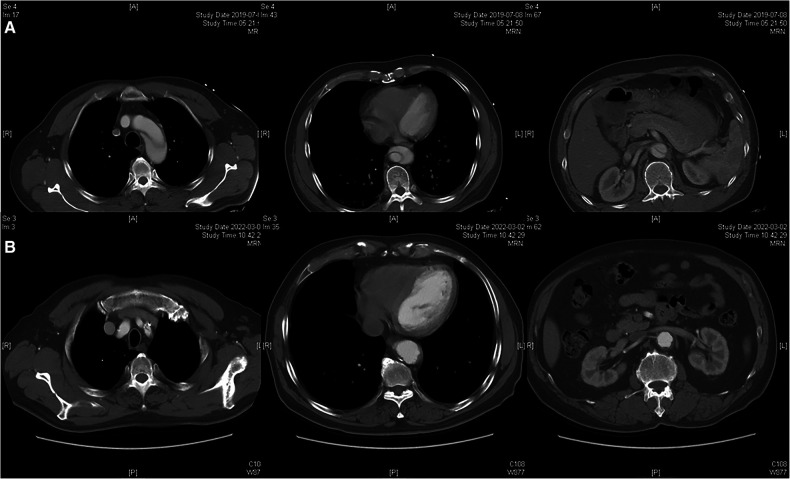
Radiographic image of fenestration technique. (**A**) Preoperative CTA showed the dissection involving the distal aortic arch and visceral aortic segment. (**B**) Postoperative CTA indicated the patency of the LSA and complete thrombosis of the visceral aortic segment over 2 years.

**Figure 4:**
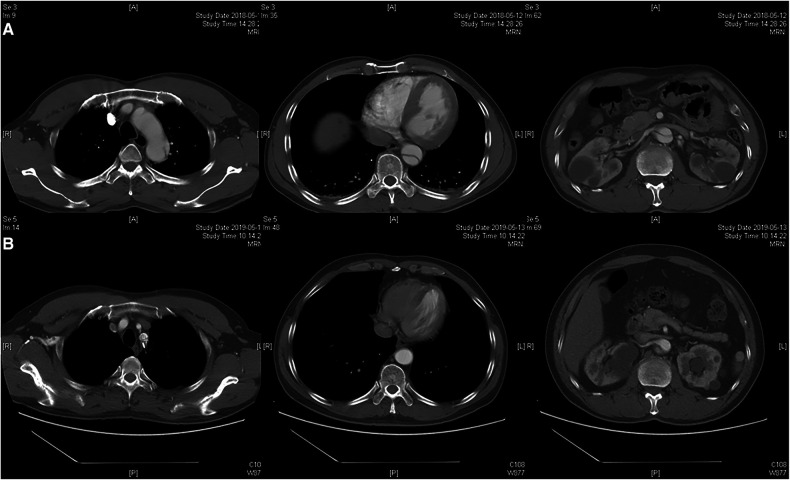
Radiographic image of chimney technique. (**A**) Preoperative CTA showed the dissection involving the distal aortic arch and visceral aortic segment. (**B**) Postoperative CTA indicated the patency of the chimney stent and partial thrombosis of the visceral aortic segment during 1-year follow-up.

The follow-up protocols, including clinical and radiographic surveillance, were performed for all patients before discharge, at 3 and 6 months after the procedure, and yearly thereafter (Figures 3, 4).

### Statistical analysis

Continuous variables were expressed as mean ± standard deviation and analyzed using Student's *t*-test. Comparisons of categorical variables were performed using the Pearson *χ*^2^ test, continuity-corrected *χ*^2^ test, or Fisher's exact test. Kaplan-Meier curves were calculated when reporting rupture-free survival and LSA patency. The follow-up period was dated to the last clinical and radiographic examination. Statistical significance was set at *p* value <0.05. The analysis was performed using SPSS version 19 (IBM Corp., Armonk, NY, USA).

## Results

The demographic characteristics of patients are presented in Table 1. The mean age was 54.54 and 53.98 years in groups A and B, and the majority of patients in both groups were male and had a history of hypertension. In group A, there was one patient with atrial septal defect, one patient with cerebral hemorrhage, and one patient with a left renal stone. In group B, one patient had renal atrophy, one patient had cerebral infarction, and two patients had abdominal aortic aneurysms. No significant difference in preoperative comorbidities was detected between the two groups. The parameters of the thoracic aorta pathologies that exhibited no significant difference between the two groups are also described in Table 1.

**Table 1:** Baseline characteristics of patients.

	Fenestration (*n* = 41)	Chimney (*n* = 42)	*p*
Age, year	54.54 ± 11.49	53.98 ± 13.67	0.84
Gender, m	29	31	0.754
Co-morbidity, *n*			
Hypertension	35	36	0.964
CAD	1	1	1
DM	0	2	0.494
Others	3	4	1
Parameters of the thoracic aorta			
Zone 2 diameter, mm	31.05 ± 3.07	30.40 ± 2.91	0.329
Length of the proximal neck, mm	9.61 ± 3.17	9.33 ± 3.32	0.699
Distal attachment zone diameter, mm	23.44 ± 2.97	23 ± 3.19	0.518
Confined to thoracic aorta, *n*	10	11	0.85
Extend proximal to LSA, *n*	6	8	0.591

CAD, coronary artery disease; DM, diabetes mellitus; LSA, left subclavian artery.

Perioperative details are shown in Table 2. The technical success rates were 92.68% and 97.62% in groups A and B, respectively. Except for blood loss, the remaining secondary technical endpoints, including procedure time, fluoroscopy time, contrast load, and hospital length of stay, showed no significant difference between the two groups. TEVAR-related death was found in four patients, two in each group. Immediate post-procedural endoleaks were detected in two (type I) and three patients (one type I and two type II) in groups A and B, respectively. Neither spinal cord ischemia nor stroke was found in either group. Only one transient ischemic attack occurred in group A, and it resolved spontaneously before discharge. The parameters of stent-graft, number of stent-grafts, oversize, coverage length, and distance to the proximal end of the aortic graft trunk were not significantly different between the two groups.

**Table 2:** Peri-operative outcome.

	Fenestration (*n* = 41)	Chimney (*n* = 42)	*p*
Technical success, *n* (%)	38 (92.68)	41 (97.62)	0.591
Secondary technical end points			
Procedure time, minutes	115.37 ± 28.64	117.38 ± 31.38	0.761
Fluoroscopy time, minutes	20.12 ± 3.79	20.57 ± 4.34	0.617
Blood loss, ml	25.73 ± 8.56	31.55 ± 8.94	0.003
Contrast load, ml	104.02 ± 9.50	108.45 ± 12.27	0.07
Hospital length of stay, days	15.29 ± 3.12	16.38 ± 6.02	0.306
TEVAR-related death, *n* (%)	2 (4.88)	2 (4.76)	1
Complications, *n* (%)			
Immediate endoleak	2 (4.88)	3 (7.14)	1
Spinal cord ischemia	0	0	N/A
Stroke	0	0	N/A
Others	3 (7.32)	3 (7.14)	1
Combined complications	5 (12.20)	6 (14.29)	0.779
Parameters of stent-graft			
Numbers of stent-graft, *n*	42	45	
Oversize, %	5.16 ± 1.84	5.06 ± 1.88	0.811
Coverage length, mm	195.61 ± 8.38	201.67 ± 18.73	0.062
Distal to the proximal end, mm	8.14 ± 3.38	9.10 ± 1.96	0.12

TEVAR, thoracic endovascular aortic repair.

**Figure 5:**
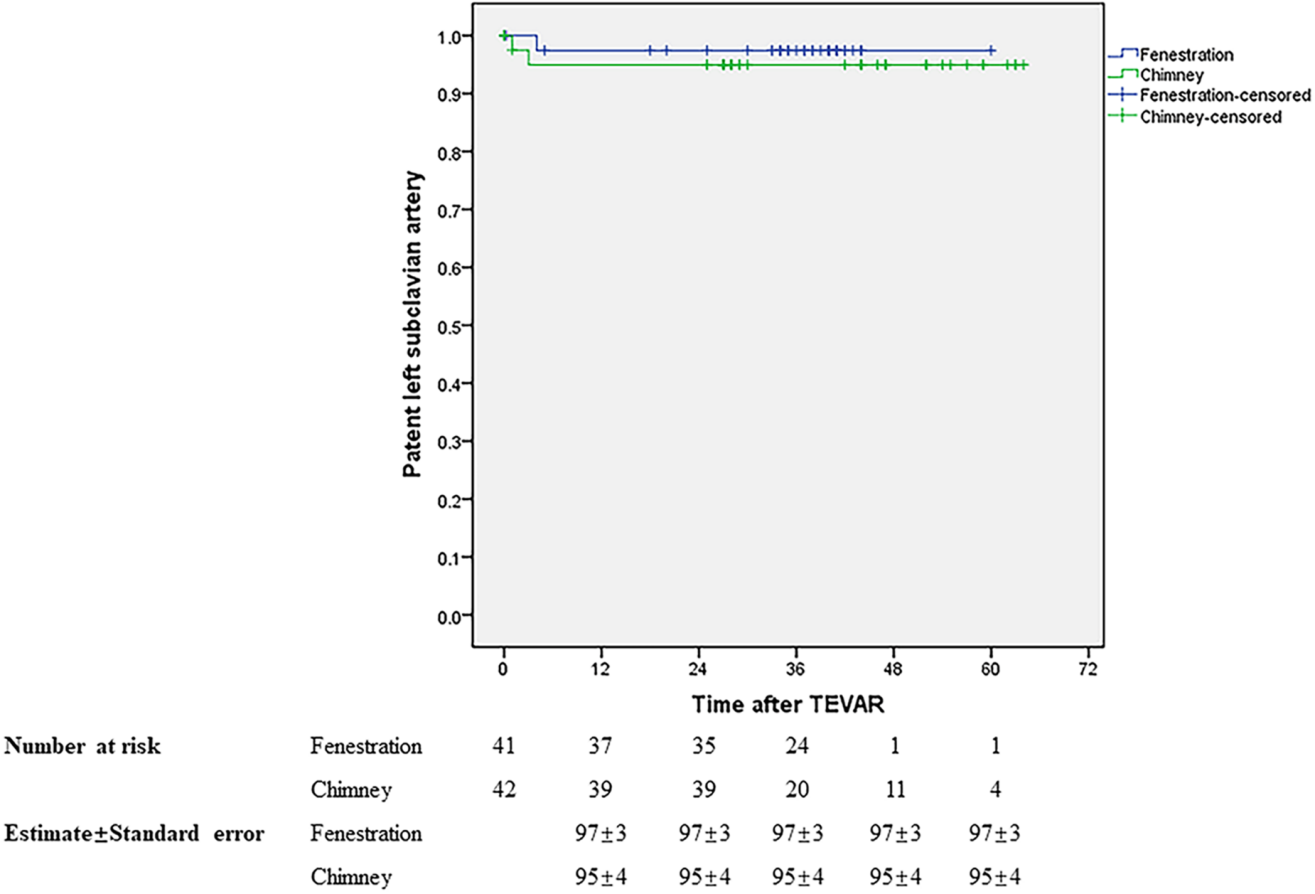
Rupture-free survival during the mid-term follow-up.

**Figure 6:**
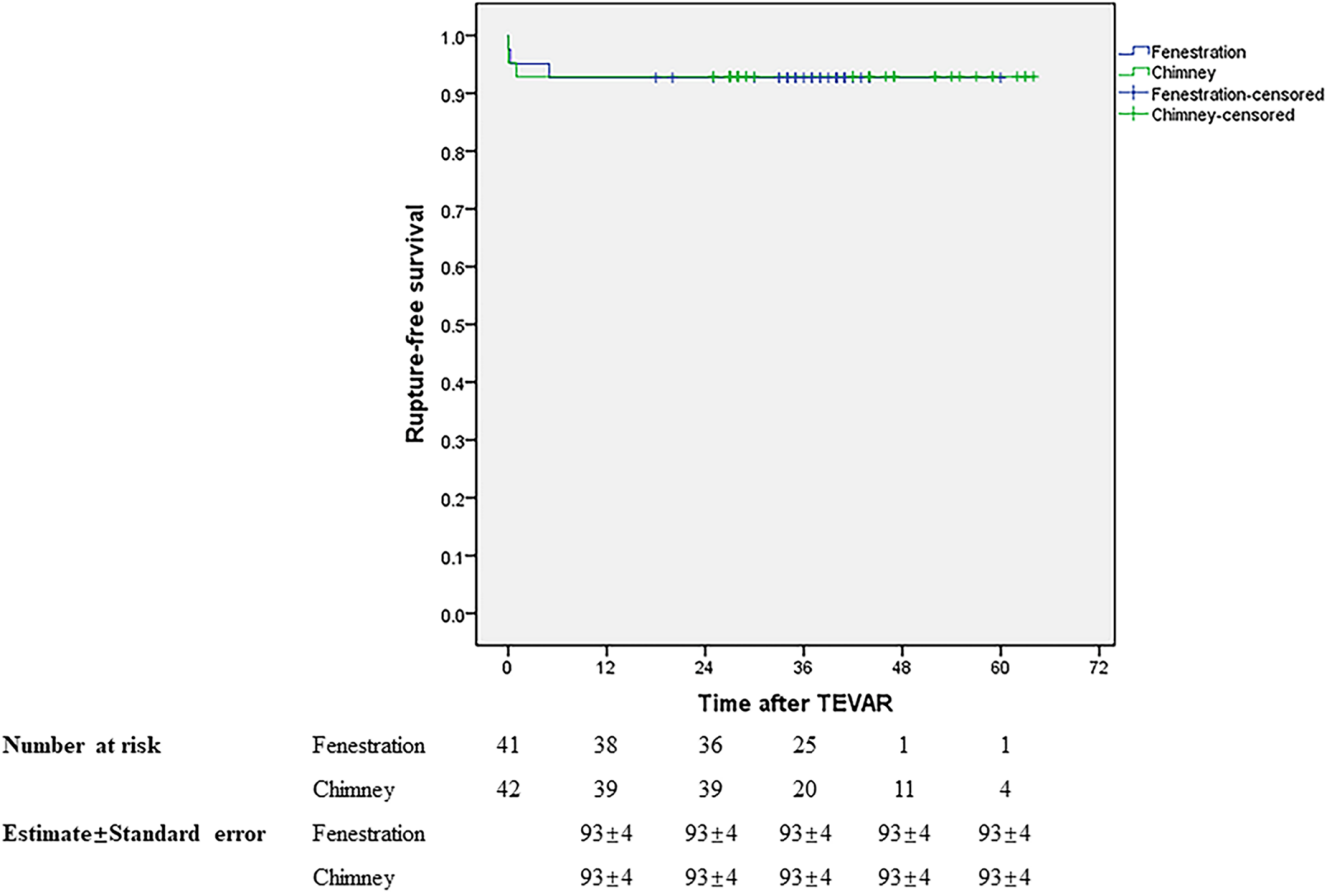
LSA patency during the mid-term follow-up.

Initial clinical success was achieved in 37 (90.24%) and 39 (92.86%) patients in groups A and B, respectively. During a mean follow-up of 34.88 months in groups A, the primary and secondary mid-term clinical success rates were 87.5% and 90%. Both of them were 92.68% in group B with a mean follow-up of 37.49 months. The mid-term primary and secondary clinical success rates showed no significant difference between the two groups, similar to the short-term outcomes. Table 3 presents the results. During follow-up, one patient died in month 5 due to lung cancer in group A and one patient died 1 month later due to acute upper gastrointestinal bleeding in group B. The LSA was patent in 37 and 38 patients in groups A and B, respectively. No stroke occurred in either group during follow-up. There was no significant difference in rupture-free survival and LSA patency between the two groups during the mid-term follow-up (Figures 5, 6).

**Table 3:** The primary and secondary endpoints.

	Fenestration (*n* = 41)	Chimney (*n* = 42)	*p*
Initial clinical success, *n*	37	39	0.973
Primary clinical success, *n*			
Short-term	35	38	0.682
Mid-term	35	38	0.682
Secondary clinical success, *n*			
Short-term	36	38	0.973
Mid-term	36	38	0.973
Endoleak, *n*			
Type I or III	2	1	0.983
Type II	0	2	0.494
Stroke	0	0	N/A
Rupture-free survival, *n*	38	39	0.984
LSA patency, *n*	37	38	0.553

LSA, left subclavian artery.

Remodeling of the aorta is shown in Table 4. According to the last CTA, complete thrombosis of the false lumen in the aorta distal to the stent graft was confirmed in 23 and 22 patients in groups A and B, and partial thrombosis was confirmed in 11 and 14 patients at the same level. The incidence of complete thrombosis in the aorta distal to the stent graft was 67.65% and 61.11% in groups A and B, respectively. Eight patients with complete thrombosis and 11 patients with partial thrombosis were detected in the visceral aortic segment in group A, and 10 patients with complete thrombosis and 11 patients with partial thrombosis were found in the visceral aortic segment in group B. The incidence of partial and complete thrombosis of false lumens significantly increased after TEVAR in both groups. Stable and reduced transaortic diameter of the aorta distal to the stent graft and visceral aortic segment were observed in the majority of patients in both groups. No significant enlargement of the false lumen was observed during follow-up. Both physician-modified techniques significantly promoted favorable aortic remodeling with negligible differences.

**Table 4:** Aortic remodeling during mid-term follow-up.

	Fenestration		Chimney	
	Pre-TEVAR	Post-TEVAR	Pre-TEVAR	Post-TEVAR
Aorta distal to SG				
Complete thrombosis, *n*	0	23	0	22
Partial thrombosis, *n*	4	11	7	14
Patent, *n*	30	0	29	0
*p* *	<0.001		<0.001	
Significant reduction, *n*	10		12	
Significant enlargement, *n*	0		0	
No significant change, *n*	24		23	
*p* **	0.664			
Visceral aortic segment				
Complete thrombosis, *n*	0	8	0	10
Partial thrombosis, *n*	4	11	5	11
Patent, *n*	20	5	20	4
*p* *	<0.001		<0.001	
Significant reduction, *n*	4		6	
Significant enlargement, *n*	0		0	
No significant change, *n*	20		19	
*p* **	0.523			

SG, stent graft; TEVAR, thoracic endovascular aortic repair.

*p, pre. vs. post.

**p, fenestration vs. chimney.

During follow-up, three complications, including one wound infection, one pulmonary infection, and one retrograde type A dissection (RTAD), were detected in group A. One case of renal insufficiency, one case of celiac thrombosis, and one case of upper extremity ischemia were found in group B. Except for the requirement of open repair for RTAD in group A, the remaining complications resolved with nominal intervention among the two groups.

The last CTA confirmed two residual type II endoleaks during follow-up. However, no re-intervention was required due to clinical silence and no significant enlargement in the false lumen. During follow-up, three residual type I endoleaks disappeared spontaneously (two at 18 months and one at 24 months). No other major complications were detected in either group during follow-up.

## Discussion

TEVAR using zone 2 as a proximal landing zone has been performed for pathologies involving the distal aortic arch (17–19). Additionally, several commercially available devices and physician-modified techniques, including single-branched stent-graft, fenestration and chimney technique, and carotid-subclavian artery bypass, have been employed to keep the LSA patent to decrease the risk of posterior stroke and upper extremity ischemia (20–22). However, neither randomized controlled studies nor guidelines have been introduced for choosing different techniques for zone 2 TEVAR. In the present study, we compared fenestration technique with chimney technique for LSA revascularization during zone 2 TEVAR, and introduced our experience.

Although the in-situ fenestration technique is more prevalent in fenestrated zone 2 TEVAR (23, 24), all patients in the present study were treated with the on-the-table fenestration technique according to our experience and previous reports (16, 21, 25). According to previous studies, the technical success rate of fenestration ranges from 90% to 100%, with no significant difference between *in situ* and on-the-table techniques (8, 21, 23, 25). Similar to previous reports, technical success was achieved in 38 (92.68%) patients in group A, and only one LSA occlusion was detected during the mid-term follow-up. Creation of the fenestration in line with the radiopaque middle-8-marker on the proximal end of the stent graft and minor rotation to adjust the fenestration orientation during the procedure are associated with a satisfactory technical success rate and high LSA patency.

Both covered and bare stents are used as chimney stents for LSA revascularization during zone 2 TEVAR (6, 26, 27). According to a previous report, covered stents had better primary patency rates than bare metal stents in aortoiliac occlusive disease (14). Endovascular treatment with primary stenting for LSA stenotic and occlusive lesions results in acceptable long-term patency with a decreased risk of perioperative complications. However, a comparison between different stents has not been performed (28). Currently, neither guidelines nor randomized controlled studies have been performed to establish selection criteria for chimney stents. Despite the use of only self-expanded bare metal stents as chimney stents in our study, LSA patency was achieved in 38 (90.48%) patients during mid-term follow-up, which was comparable to the results in previous reports (27, 29).

Carotid to subclavian bypass has been considered as the standard treatment for LSA revascularization during zone 2 TEVAR (30). However, surgical debranching carries 29% of the early complications, including stroke, phrenic nerve palsy, hematoma, and chyle leak (30, 31). Carotid to subclavian artery bypass was not routinely considered during zone 2 TEVAR at our center. Therefore, a comparison between endovascular repair and hybrid surgery was not conducted.

During the mid-term follow-up, three immediate postoperative type I endoleaks in both groups disappeared spontaneously. Hemodynamic and anatomical changes after stent graft deployment may contribute to false lumen thrombosis and promote favorable aortic remodeling. Two type II endoleaks remained patent in group B during follow-up, and the gutter arising between the proximal landing zone and the stent-graft is considered to be related to this dilemma (32). No major neurological complications were found in our study. LSA revascularization and limited coverage of the thoracic segmental arteries were related to a decreased risk of stroke and spinal cord ischemia (33).

Both techniques contributed significantly to the favorable aortic remodeling with negligible differences during mid-term follow-up. A sufficient proximal seal promoted complete thrombosis of the false lumen in the distal aortic arch and stented segment of the thoracic aorta, and prevented further aortic enlargement and rupture. The variety of thrombosis of the false lumen was confirmed at the level of the aorta distal to the stent graft and visceral aortic segment during follow-up. Further thrombosis of the false lumen and aortic remodeling processes are a matter of time. Retrograde flow from distal entry tears could serve as a predictor of aortic remodeling. Moreover, the outcomes should be interpreted carefully after considering selection biases and a limited number of patients.

The present study has several limitations. First, the outcomes of this retrospective study with a limited number of patients and experience in a single center may not be generally applicable. A larger randomized controlled study with long-term follow-up is required to confirm these findings. Second, only self-expanded bare stents were used as chimney stents in zone 2 TEVAR. Comparisons with other techniques, including covered stents serving as chimney stents and carotid to subclavian artery bypass or transposition, should be performed to establish the selection criteria for choosing different techniques for LSA revascularization during zone 2 TEVAR. Third, the on-the-table modified fenestrated stent-graft required multiview fluoroscopy to confirm insertion orientation. Additionally, deployment of a stent severing as a bailout strategy to maintain the LSA patent may be required.

In conclusion, both fenestration and chimney techniques, with a significantly decreased incidence of stroke and spinal cord ischemia, are safe and feasible for LSA revascularization during zone 2 TEVAR. Minor orientation of the stent graft is difficult in a tortuous and calcified aorta and iliac artery, and a lower rate of clinical and technical success for the fenestration technique was detected. However, both techniques significantly contributed to favorable aortic remodeling during mid-term follow-up. Long-term clinical and radiographic surveillance are required to confirm these findings.

## Data Availability

The raw data supporting the conclusions of this article will be made available by the authors, without undue reservation.

## References

[1] Czerny M Pacini D Aboyans V Al-Attar N Eggebrecht H Evangelista A Current options and recommendations for the use of thoracic endovascular aortic repair in acute and chronic thoracic aortic disease: an expert consensus document of the European society for cardiology (ESC) working group of cardiovascular surgery, the ESC working group on aorta and peripheral vascular diseases, the European association of percutaneous cardiovascular interventions (EAPCI) of the ESC and the European association for cardio-thoracic surgery (EACTS). Eur J Cardiothorac Surg. (2021) 59:65–73. 10.1093/ejcts/ezaa268 33011773

[2] Riambau V Böckler D Brunkwall J Cao P Chiesa R Coppi G Editor's choice – management of descending thoracic aorta diseases. Eur J Vasc Endovasc Surg. (2017) 53:4–52. 10.1016/j.ejvs.2016.06.005 28081802

[3] Sobocinski J Patterson BO Karthikesalingam A Thompson MM. The effect of left subclavian artery coverage in thoracic endovascular aortic repair. Ann Thorac Surg. (2016) 101:810–7. 10.1016/j.athoracsur.2015.08.069 26718858

[4] Chen X Wang J Premaratne S Zhao J Zhang WW. Meta-analysis of the outcomes of revascularization after intentional coverage of the left subclavian artery for thoracic endovascular aortic repair. J Vasc Surg. (2019) 70:1330–40. 10.1016/j.jvs.2019.03.022 31176636

[5] Karaolanis GI Antonopoulos CN Charbonneau P Georgakarakos E Moris D Scali S A systematic review and meta-analysis of stroke rates in patients undergoing thoracic endovascular aortic repair for descending thoracic aortic aneurysm and type B dissection. J Vasc Surg. (2022) 76:292–301.e3. 10.1016/j.jvs.2022.02.031 35248694

[6] Zhang L Wu MT Zhu GL Feng JX Song C Li HY Off-the-shelf devices for treatment of thoracic aortic diseases: midterm follow-up of TEVAR with chimneys or physician-made fenestrations. J Endovasc Ther. (2020) 27:132–42. 10.1177/1526602819890107 31789078

[7] Xue Y Sun L Zheng J Huang X Guo X Li T The chimney technique for preserving the left subclavian artery in thoracic endovascular aortic repair. Eur J Cardiothorac Surg. (2015) 47:623–9. 10.1093/ejcts/ezu266 25009212 PMC4358408

[8] Zhu J Zhao L Dai X Luo Y Fan H Feng Z Fenestrated thoracic endovascular aortic repair using physician modified stent grafts for acute type B aortic dissection with unfavourable landing zone. Eur J Vasc Endovasc Surg. (2018) 55:170–6. 10.1016/j.ejvs.2017.11.012 29241685

[9] Patel HJ Dake MD Bavaria JE Singh MJ Filinger M Fischbein MP Branched endovascular therapy of the distal aortic arch: preliminary results of the feasibility multicenter trial of the gore thoracic branch endoprosthesis. Ann Thorac Surg. (2016) 102:1190–8. 10.1016/j.athoracsur.2016.03.091 27262912

[10] van Bakel TM de Beaufort HW Trimarchi S Marrocco-Trischitta MM Bismuth J Moll FL Status of branched endovascular aortic arch repair. Ann Cardiothorac Surg. (2018) 7:406–13. 10.21037/acs.2018.03.13 30155420 PMC6094020

[11] Li C Xu P Hua Z Jiao Z Cao H Liu S Early and midterm outcomes of in situ laser fenestration during thoracic endovascular aortic repair for acute and subacute aortic arch diseases and analysis of its complications. J Vasc Surg. (2020) 72:1524–33. 10.1016/j.jvs.2020.01.072 32273224

[12] Liu F Zhang W Wang G Yuan T Shu X Guo D Long-term outcomes of balloon-expandable bare stent as chimney stent in thoracic endovascular aortic repair for supra-aortic branches reconstruction. J Thorac Dis. (2019) 11:1261–8. 10.21037/jtd.2019.04.15 31179068 PMC6531743

[13] Kanaoka Y Ohki T Maeda K Baba T. Analysis of risk factors for early type I endoleaks after thoracic endovascular aneurysm repair. J Endovasc Ther. (2017) 24:89–96. 10.1177/1526602816673326 27760812

[14] Society for Vascular Surgery Lower Extremity Guidelines Writing Group, Conte MS Pomposelli FB Clair DG Geraghty PJ McKinsey JF Mills JL Society for vascular surgery practice guidelines for atherosclerotic occlusive disease of the lower extremities: management of asymptomatic disease and claudication. J Vasc Surg. (2015) 61:2S–41S. 10.1016/j.jvs.2014.12.009 25638515

[15] Fillinger MF Greenberg RK McKinsey JF Chaikof EL, Society for Vascular Surgery Ad Hoc Committee on TRS. Reporting standards for thoracic endovascular aortic repair (TEVAR). J Vasc Surg. (2010) 52:1022–33; 1033 e15. 10.1016/j.jvs.2010.07.008 20888533

[16] Chang H Wang Y Liu B Wang W Li Y. Endovascular repair for acute type B aortic dissection with unfavorable proximal landing zone. Ann Thorac Surg. (2022) 113:545–53. 10.1016/j.athoracsur.2021.02.092 33819473

[17] Johnson CE Zhang L Magee GA Ham SW Ziegler KR Weaver FA Periscope sandwich stenting as an alternative to open cervical revascularization of left subclavian artery during zone 2 thoracic endovascular aortic repair. J Vasc Surg. (2021) 73:466.e3–75.e3. 10.1016/j.jvs.2020.05.063 32622076

[18] Miura S Kurimoto Y Maruyama R Wada T Konno M Iba Y Thoracic endovascular aortic repair on zone 2 landing for type B aortic dissection. Ann Vasc Surg. (2019) 60:120–7. 10.1016/j.avsg.2019.02.017 31075454

[19] Bradshaw RJ Ahanchi SS Powell O Larion S Brandt C Soult MC Left subclavian artery revascularization in zone 2 thoracic endovascular aortic repair is associated with lower stroke risk across all aortic diseases. J Vasc Surg. (2017) 65:1270–9. 10.1016/j.jvs.2016.10.111 28216353

[20] Jing Z Lu Q Feng J Zhou J Feng R Zhao Z Endovascular repair of aortic dissection involving the left subclavian artery by castor stent graft: a multicentre prospective trial. Eur J Vasc Endovasc Surg. (2020) 60:854–61. 10.1016/j.ejvs.2020.08.022 33183920

[21] Chassin-Trubert L Mandelli M Ozdemir BA Alric P Gandet T Canaud L. Midterm follow-up of fenestrated and scalloped physician-modified endovascular grafts for zone 2 TEVAR. J Endovasc Ther. (2020) 27:377–84. 10.1177/1526602819881128 31645219

[22] Pecoraro F Lachat M Cayne NS Pakeliani D Rancic Z Puippe G Mid-term results of chimney and periscope grafts in supra-aortic branches in high risk patients. Eur J Vasc Endovasc Surg. (2017) 54:295–302. 10.1016/j.ejvs.2017.06.014 28754428

[23] Zhao Z Qin J Yin M Liu G Liu X Ye K In situ laser stent graft fenestration of the left subclavian artery during thoracic endovascular repair of type B aortic dissection with limited proximal landing zones: 5-year outcomes. J Vasc Interv Radiol. (2020) 31:1321–7. 10.1016/j.jvir.2020.02.025 32684418

[24] Sonesson B Dias N Abdulrasak M Resch T. Midterm results of laser generated in situ fenestration of the left subclavian artery during thoracic endovascular aneurysm repair. J Vasc Surg. (2019) 69:1664–9. 10.1016/j.jvs.2018.09.052 30591297

[25] Kuo HS Huang JH Chen JS. Handmade fenestrated stent grafts to preserve all supra-aortic branches in thoracic endovascular aortic repair. J Thorac Cardiovasc Surg. (2020) 160:629.e1–39.e1. 10.1016/j.jtcvs.2019.07.096 31564542

[26] Carter R Wee IJY Petrie K Syn N Choong AMTL. Chimney parallel grafts and thoracic endovascular aortic repair for blunt traumatic thoracic aortic injuries: a systematic review. Vascular. (2018) 27:204–12. 10.1177/1708538118812548 30522411

[27] Ramdon A Patel R Hnath J Yeh CC Darling RC 3rd. Chimney stent graft for left subclavian artery preservation during thoracic endograft placement. J Vasc Surg. (2020) 71:758–66. 10.1016/j.jvs.2019.05.049 32089209

[28] Soga Y Tomoi Y Fujihara M Okazaki S Yamauchi Y Shintani Y Perioperative and long-term outcomes of endovascular treatment for subclavian artery disease from a large multicenter registry. J Endovasc Ther. (2015) 22:626–33. 10.1177/1526602815590579 26092540

[29] Voskresensky I Scali ST Feezor RJ Fatima J Giles KA Tricarico R Outcomes of thoracic endovascular aortic repair using aortic arch chimney stents in high-risk patients. J Vasc Surg. (2017) 66:9.e3–20.e3. 10.1016/j.jvs.2016.11.063 28216358 PMC5483394

[30] Voigt SL Bishawi M Ranney D Yerokun B McCann RL Hughes GC. Outcomes of carotid-subclavian bypass performed in the setting of thoracic endovascular aortic repair. J Vasc Surg. (2019) 69:701–9. 10.1016/j.jvs.2018.07.022 30528402

[31] Bianco V Sultan I Kilic A Aranda-Michel E Cuddy RJ Srivastava A Concomitant left subclavian artery revascularization with carotid-subclavian transposition during zone 2 thoracic endovascular aortic repair. J Thorac Cardiovasc Surg. (2020) 159:1222–7. 10.1016/j.jtcvs.2019.03.060 31030960

[32] Shu C Fan B Luo M Li Q Fang K Li M Endovascular treatment for aortic arch pathologies: chimney, on-the-table fenestration, and in-situ fenestration techniques. J Thorac Dis. (2020) 12:1437–48. 10.21037/jtd.2020.03.10 32395281 PMC7212147

[33] Czerny M Schmidli J Adler S van den Berg JC Bertoglio L Carrel T Current options and recommendations for the treatment of thoracic aortic pathologies involving the aortic arch: an expert consensus document of the European association for cardio-thoracic surgery (EACTS) and the European society for vascular surgery (ESVS). Eur J Cardiothorac Surg. (2019) 55:133–62. 10.1093/ejcts/ezy313 30312382

